# Integrating real‐time in vivo tumour genomes for longitudinal analysis and management of glioma recurrence

**DOI:** 10.1002/ctm2.567

**Published:** 2021-11-04

**Authors:** Zhiyuan Sheng, Jinliang Yu, Kaiyuan Deng, Yage Bu, Shuang Wu, Sensen Xu, Yushuai Gao, Qianqian Zhang, Zhaoyue Yan, Chaojie Bu, Zhongcan Chen, Jianjun Gu, Yan Jia, Xinya Gao, Ajmal Zemmar, Fitri Sumardi, Juha Hernesniemi, Lingfei Kong, Gang Liu, Ming Li, Meiyun Wang, Tianxiao Li, Xingyao Bu

**Affiliations:** ^1^ Department of Neurosurgery Zhengzhou University People's Hospital Henan Provincial People's Hospital Zhengzhou China; ^2^ Juha International Center for Neurosurgery Henan Provincial People's Hospital Zhengzhou China; ^3^ Juha International Central Laboratory of Neurosurgery Henan Provincial People's Hospital Zhengzhou China; ^4^ Department of Neurology Henan Provincial People's Hospital Zhengzhou China; ^5^ Laboratory of Neurology Henan Provincial People's Hospital Zhengzhou China; ^6^ Department of Radiology Henan Provincial People's Hospital Zhengzhou China; ^7^ Department of Pathology Henan Provincial People's Hospital Zhengzhou China; ^8^ Department of Center for Clinical Single Cell Biomedicine Clinical Research Center Department of Oncology Henan Provincial People's Hospital The People's Hospital of Zhengzhou University Zhengzhou China

Dear Editor,

Tumour recurrence is the leading cause of glioma mortality. Current therapies based on excised tumour tissues cannot be lastingly effective due to the waning representativeness of in vitro primary tumours for the in vivo glioma caused by the spatiotemporal heterogeneity. Therefore, real‐time in vivo genomic information of the tumour is needed for precision management and longitudinal analysis of glioma recurrence. We here evaluated the clinical applicability of glioma‐derived circulating tumour DNA (ctDNA) from cerebrospinal fluid (CSF) and tumour in situ fluid (TISF), the latter denoting the fluid within surgical cavities,^1^ and proposed the CSF‐tumour tissue‐TISF (CTT) sequencing pattern that allows in vivo real‐time genomic profiling of glioma during the clinical course.

We detected ctDNA in TISF from 36 of 36 (100%) patients during their post‐operative courses. These gliomas covered different subtypes, locations and progression stages (0.3‐42.4 months after primary surgery) (Table S1). Importantly, we found that variant allele fractions (VAFs) of TISF ctDNA increased with tumour progression, even when the process is beyond imaging modalities (Figure 1A, Figure S1A,B). Moreover, the TISF ctDNA detected before post‐operative anti‐tumour treatment probably mirrored the molecular residue and complemented tumour tissues for the spatial architecture of the genome of primary gliomas (Figure 1B).

**FIGURE 1:**
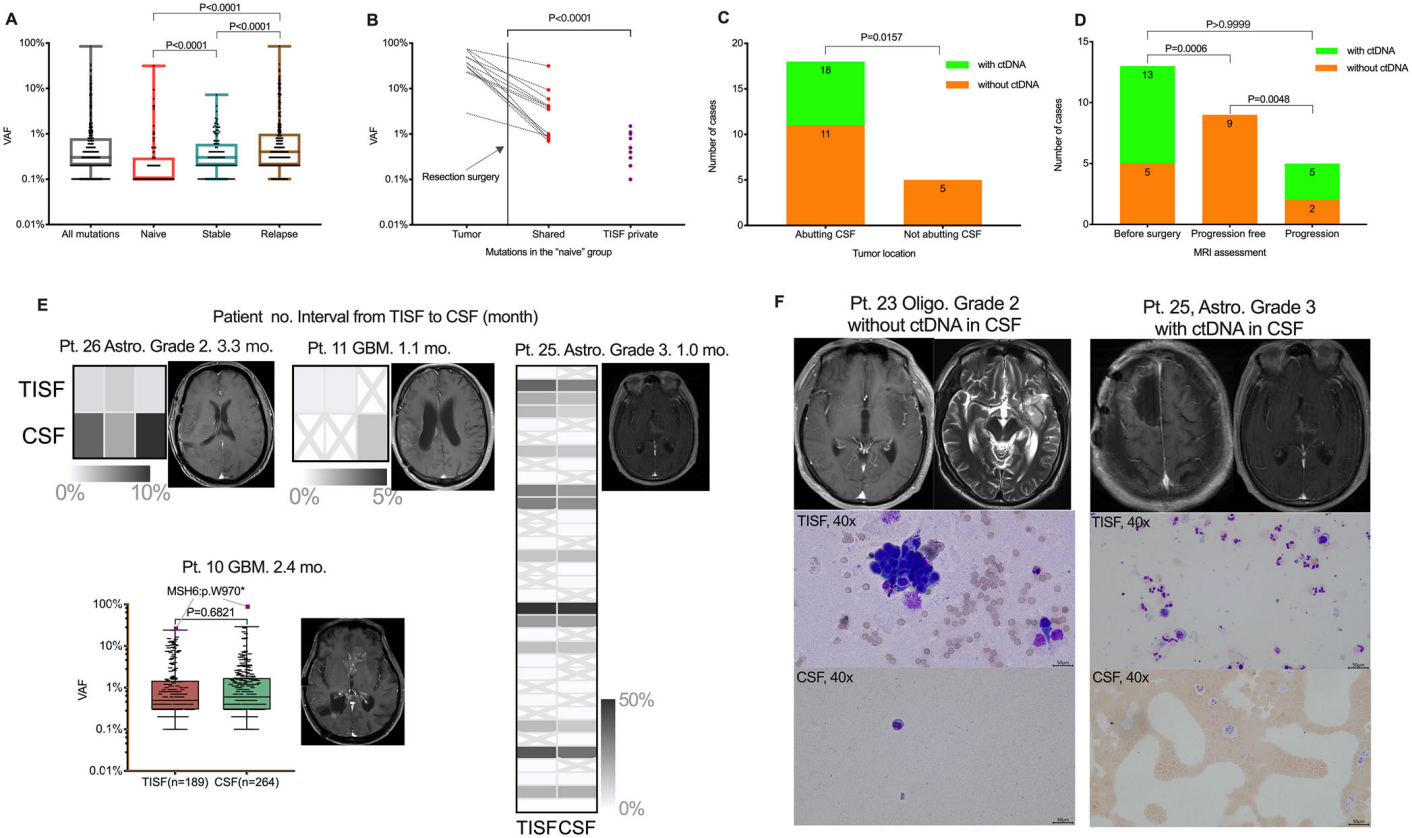
Circulating tumour DNA and malignant cells in tumour in situ fluid (TISF) and cerebrospinal fluid (CSF). (A) Variant allele fractions (VAFs) of mutations in the TISF. Thirty‐six patients were divided into three groups: 'Naive' included six patients who had not received radiation and chemotherapy after surgery, 'stable' contained 14 patients who were at the progression‐free stage on MRI, and 'relapse' referred to 16 patients who suffered tumour progression or relapse on MRI. *p* values using the Mann–Whitney test were significant (all *p *< 0.0001) for the comparison between any two of the groups. (B) We further analyzed genetic alterations in the 'naive' group. Matched tissue‐TISF (*n* = 6) shared 12 mutations, and the remaining 108 were private to the TISF. VAFs of shared mutations were statistically higher than that of TISF private alternations (*p* < 0.0001, Mann–Whitney test). The shared mutations probably represented the residue of the resected tumour, and the TISF private ones could contribute to the spatial genomic architecture of primary gliomas. (C and D) In our cohort, detectable CSF ctDNA was associated with radiographic features including tumour touching the CSF space (*p* = 0.0048) and tumour progression (*p* < 0.005, Mann–Whitney test). (E) TISF and CSF ctDNA displayed similar genomic profiles of glioma in four patients. The MRIs were taken at the CSF collection. The heat maps showed the similarity of mutations in matched TISF and CSF. For the patient 10, 189 and 264 mutations were detected in the TISF and the CSF, respectively. The overall VAFs in both samples were statistically comparable (*p* = 0.6821, Mann–Whitney test). The seeming divergences in the two samples might be caused by the time lapse and mismatch repair (MMR) function deficiency (also see Figure 3). “X” in the boxes means 'not detected'. (F) Malignant cells in the TISF and CSF of representative cases. MRIs were taken at the cytologic analysis. Abbreviations: Astro, astrocytoma; GBM, glioblastoma; Oligo, oligodendroglioma

Tumour‐derived genetic mutations were identified in 18 of 34 CSF samples (54.5%) from 33 patients (Table S2). In our cohort, detectable CSF ctDNA was associated with radiographic features including tumour progression (*p* = 0.0048) and tumour touching the CSF space (*p* < 0.005) (Figure 1C,D). CSF ctDNA was especially insensitive to post‐operative gliomas without radiographic progression (0/9, 0%, Figure 1D). Similarly, Miller et al previously reported that shedding tumour DNA into the CSF was related to tumour progression, tumour burden and tumour spreading towards the CSF circulating system.^2^

We compared the sequencing results of 11 TISF‐CSF sample pairs from the above‐described patients (Table S3). CSF ctDNA was detected for only four of 11 patients (36.4%), all four tumours having the tumour touching CSF with radiographically seeable burdens (Figure 1E). Further analysis revealed that genetic landscapes in each TISF‐CSF paris were similar, while the seeming divergence might be accounted for by other mutagenic factors, for example, time lapse and mismatch repair (MMR) function deficiency (Figure 1E). Moreover, we found that malignant cells more likely existed in the TISF than in the CSF (5/11 vs. 0/11, *p* = 0.0351, Figure 1F).

Further analysis of TISF ctDNA revealed a broad spectrum of single nucleotide alterations. The most frequently observed alternations in the TISF were NF1 and TP53. The most frequently implicated oncogenic signaling pathway was the PI3K/RTK/RAS pathway (86.1%) (Figure S2). We did not detect the isocitrate dehydrogenase (IDH) mutation in the TISF for all IDH wild‐type gliomas (20/20, 100%), while the IDH alteration was identified in the TISF of 12 of 16 (75%) patients with IDH‐mutant glioma (Figure 2A). In addition, some treatment‐related mutations were observed in the TISF cohort. For example, temozolomide (TMZ)‐induced MMR gene mutations^3^ (MLH1, MSH2, MSH6, PMS2) were observed in nine patients (Figure S2).

**FIGURE 2:**
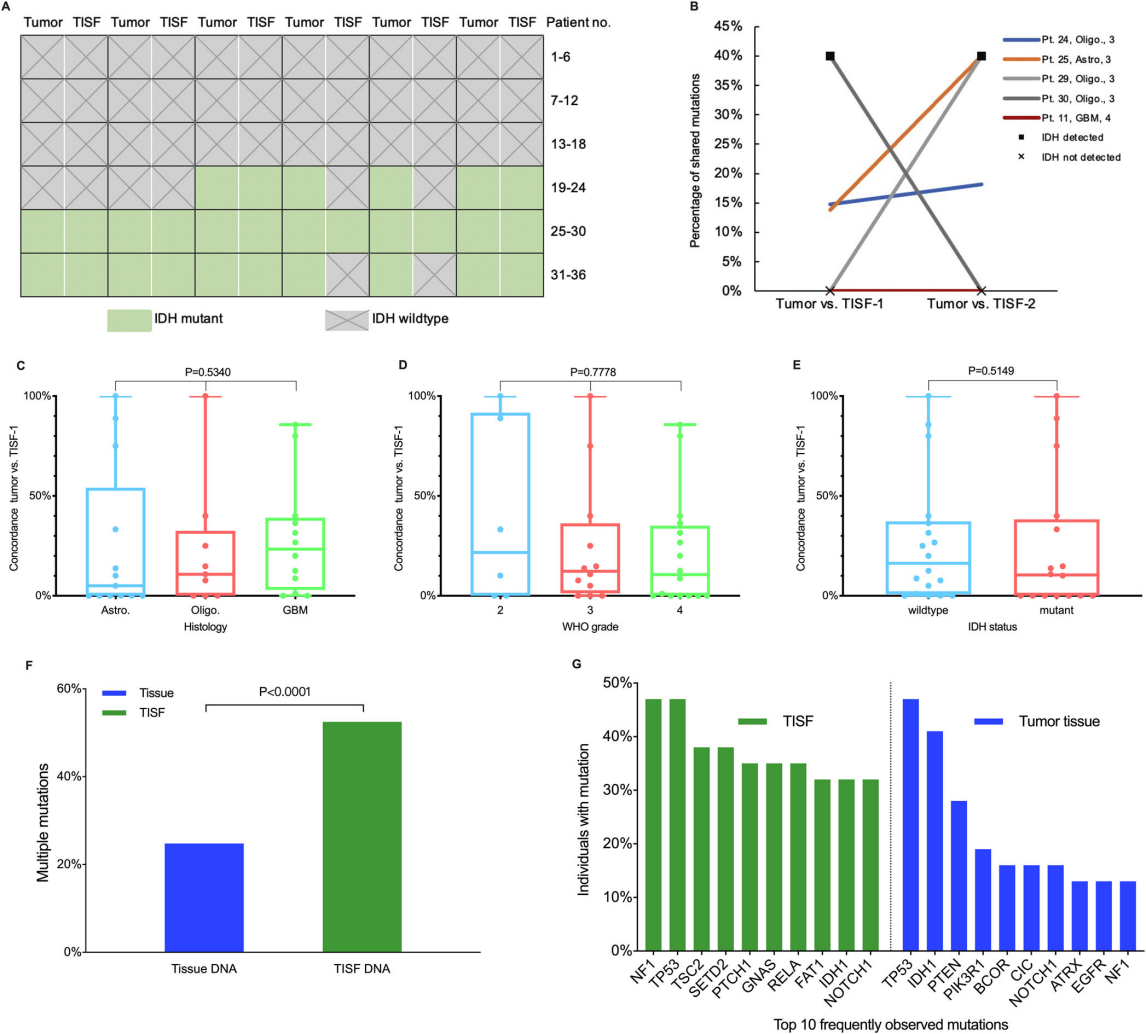
Comparisons of tumour DNA between tumour in situ fluid (TISF) and tumour tissue. (A) Comparison of IDH status in tumour and TISF. (B) Changes in frequencies of shared mutations in the serial TISF for five patients. The second TISF sample of three patients (25, 26, 29) saw an increase in the percentage of shared mutations. For patient 29, the IDH status in the TISF dynamically changed from not‐detected to mutant and from mutant to not‐detected for patient 30. (C‐E) The numerical concordance of detected mutations between the first TISF and the tumour tissue varied considerably (0%–100%, median 10.5%) without superficial clinical relevance. (F) Alternations in TISF ctDNA harbored more multiple mutations within individual oncogenes (52.5% vs. 24.8%, *p* < 0.0001, chi‐square test), this might confer enhanced oncogenicity to the recurrent glioma.^4^ (G) Respective top 10 mutations in the TISF and the tumour tissue, this transformation implied the mutable genetic path to recurrence of glioma

Comparison of tumour DNA from 34 matched TISF‐tumour pairs revealed huge dynamic heterogeneity during glioma evolution. Overall, the numerical concordance rate of detected mutations between the first TISF and the tumour tissue varied appreciably from 0%–100% (median 10.5%) without superficial clinical relevance (Figure 2C‐E). The second TISF samples of three of five patients who had repeated TISF collections saw an increase in the percentage of shared mutations (Figure 2B). TISF owned a more abundant spectrum of mutations than tumour tissues (Figure S3). Mutations in TISF ctDNA harbored more multiple mutations within individual oncogenes (Figure 2F), which might confer enhanced oncogenicity to the cancer^4^ and account for the fact that recurrent gliomas are typically more aggressive than the primary setting. Moreover, the most frequently altered genes were also different in two samples (Figure 2G), this transformation implied the mutable genetic path to recurrence of glioma. These results indicated the complexity of the evolution of glioma genomes under natural and therapeutic pressure. The discordance also echoes the Glioma Longitudinal Analysis Consortium's reports that genomic trajectories of glioma are highly alterable and patient‐specific under therapeutic pressure.^5^

By analyzing genomic information from combined tumour tissues and liquid samples, we identified various progression patterns of glioma, such as linear clonal evolution (Figure 3A) and activating downstream oncogenic pathways to hasten tumour relapse (Figure 3B). In an IDH wild‐type glioblastoma, we observed that TMZ‐induced hypermutation implicated key oncogenic pathways for glioblastoma, for example, NF1 in PI3K/AKT/mTOR pathway, CDK4 in RB pathway and TP53 in P53 pathway^6^, ^7^ (Figure 3C‐E). This implied that genomic hypermutation of glioma could stochastically activate the core oncogenic pathway, which is related to the progression of the tumour. Additionally, for a patient receiving bevacizumab salvage treatment for the recurrent glioma, comparisons of cross‐treatment TISF ctDNA evidenced the temporal beneficialness of Bevacizumab for the patient at the molecular level, which was consistent with the imaging changes (Figure S1B).

**FIGURE 3:**
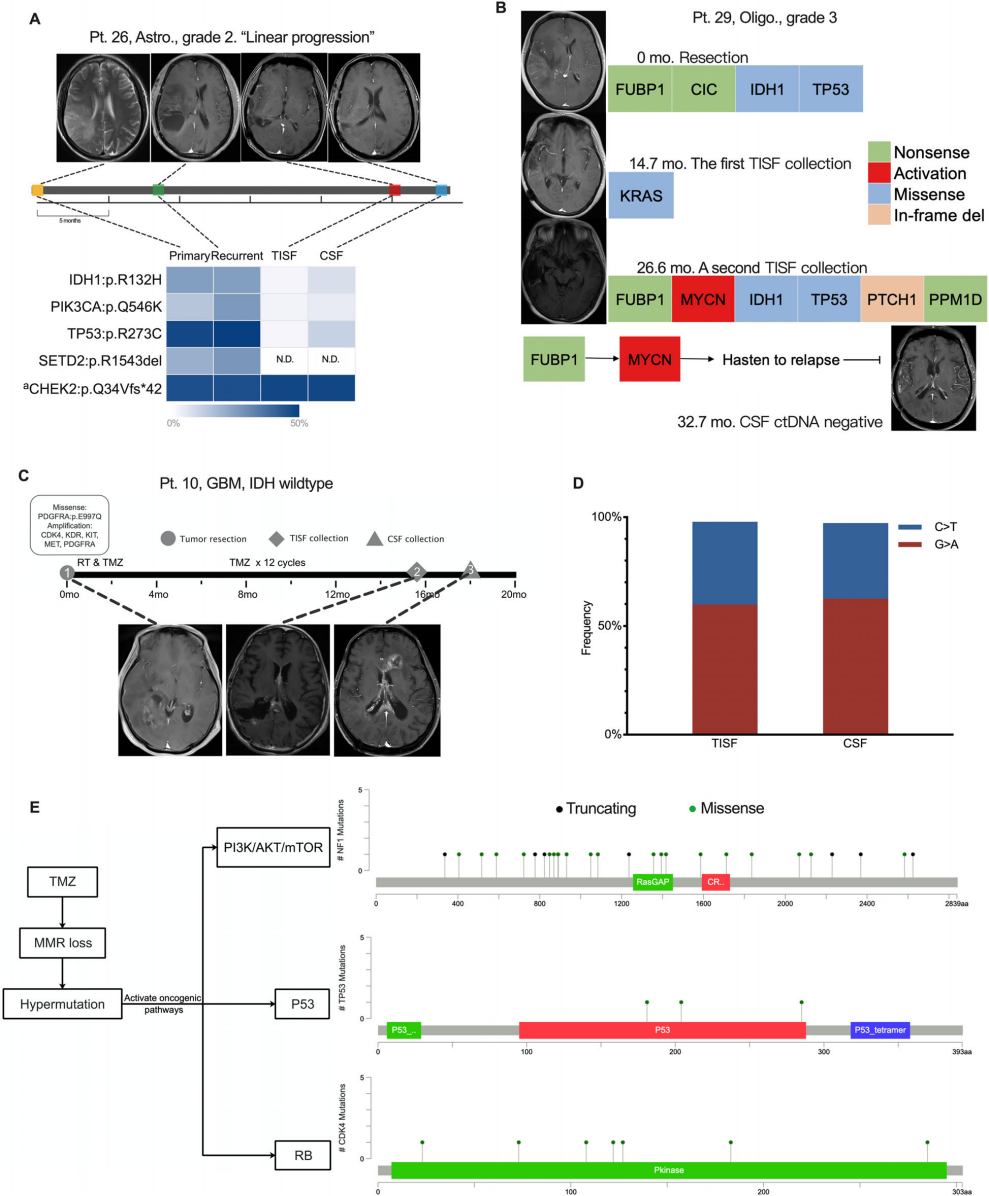
Track the evolution patterns of glioma with combined samples. (A) The glioma progressed following a linear pattern, and the slight VAF increase of mutations in the cerebrospinal fluid (CSF) compared to the tumour in situ fluid (TISF) might mirror the gradual growth of the tumour. (B) The tumour hastened to relapse with known oncogenic pathways activated. As reported, the inactivation of FUBP1 can activate the oncogene MYC in oligodendroglioma, which is related to poorer progression‐free survival,^9^, ^10^ as shown in the panel of patient 29. (C‐E) TMZ‐induced hypermutation and progression of glioblastoma. (C) The clinical course of patient 10. (D) Mutational spectrum of detected 189 and 264 genetic alterations in the TISF and the CSF, respectively. (E) Hypermutation occurred in the key oncogenic pathways for glioblastoma, that is, NF1 in PI3K/AKT/mTOR pathway, CDK4 in RB pathway, and TP53 in P53 pathway, which might be related to the progression of the tumour. The mutational maps were generated using the cBioPortal website tool. Abbreviations: Astro., astrocytoma; GBM, glioblastoma; MMR, mismatch repair; N.D., not detected; Oligo., oligodendroglioma; RT: radiation therapy; TMZ, temozolomide ^a^Germline mutation.

In the end, based on our findings and previous studies,^2^ we proposed the CTT sequencing pattern to realize the real‐time knowledge of in vivo tumour genomic information during the glioma evolution. In this pattern, CSF sequencing is mainly for preoperative diagnosis, risk stratification, providing therapeutic targets for appropriate patients, postoperative assessment of tumour relapse and dissemination and prognosis prediction.^2^ Resection surgery represents the mainstay therapy for glioma,^8^ the resultant tumour tissues are necessary for histo‐molecular diagnosis and act as the comparing baseline. And the TISF sequencing puts the glioma under routine molecular surveillance after surgery (Figure 4). Of note, TISF ctDNA is more sensitive to low tumour burden, and the procedure of TISF collection is even less invasive than the lumbar puncture, both supporting the priority of obtaining TISF over CSF, even though a glioma abuts the CSF system.

**FIGURE 4:**
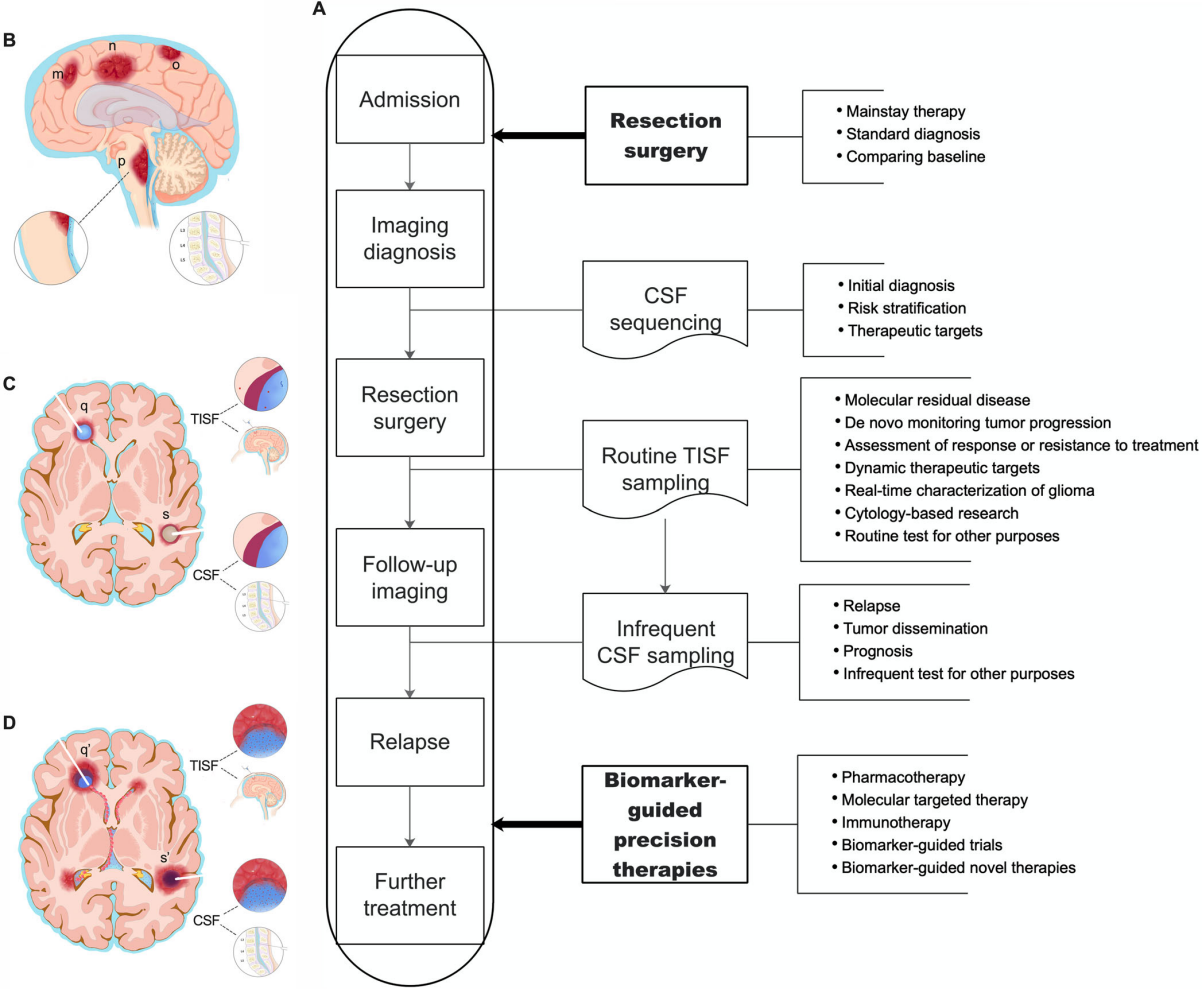
Cerebrospinal fluid‐tumour tissue‐tumour in situ fluid (CTT) sequencing pattern. (A) Flowchart of CTT pattern for precision management and monitoring of glioma. The pattern can put the glioma under molecular surveillance during the clinical course. (B), (C), and (D) are conceivable scenarios to explain the clinical applicability of tumour in situ fluid (TISF) and cerebrospinal fluid (CSF) for managing glioma. (B) The preoperative situation: The tumour **m** represents a glioma in the brain parenchyma with relatively lower burden, **n and o** stand for tumours abutting the CSF reservoir, while the tumour **p** is one located at the high‐risk region of the brainstem. The detectability of CSF ctDNA for preoperative glioma mainly depends on the tumour burden and touching CSF or not. (C) The stage of postoperatively progression‐free by imageology. Tumours **q** and **s** refer to the resected gliomas abutting or not abutting CSF, respectively. In this period, ctDNA is typically undetected in the CSF, while the routine obtaining of TISF can be utilized for the molecular monitoring of the de novo tumour recurrence. (D) means that both tumours have progressed overtly on the imaging and have certainly spread tumour DNA into the CSF. In this scenario, both samples are comparable for the real‐time in vivo genomic characterization of the glioma, while the procedure of obtaining TISF is less invasive than the lumbar puncture

In conclusion, TISF and CSF ctDNA complement tumour tissues for providing real‐time in vivo genomic profiles of glioma, especially, shedding ctDNA into TISF seems a universal property of glioma. Together, the integrated CTT pattern can realize the real‐time knowledge of in vivo glioma genomic information during the clinical course. Together with future patient‐specific multi‐omics panels, this management model should realize personalized management and longitudinal analysis of glioma recurrence.

## CONFLICT OF INTEREST

The authors have no conflict of interest to disclose.

## FUNDING INFORMATION

This work was supported by Science and Technology Tackle Program of Henan Province (grant number: 192102310126) and Joint Project of Medical Science and Technology Tackling Plan of Henan Province (grant number: 201601016).

## Supporting information

Supporting information

Supporting information

Supporting information

Supporting information

Supporting information

Supporting information
