# Supplementary figures and images for: Integrating real‐time in vivo tumour genomes for longitudinal analysis and management of glioma recurrence
Source: Clin Transl Med. 2021 Nov 4;11(11):e567. doi: 10.1002/ctm2.567 (PMC8567036; doi:10.1002/ctm2.567)

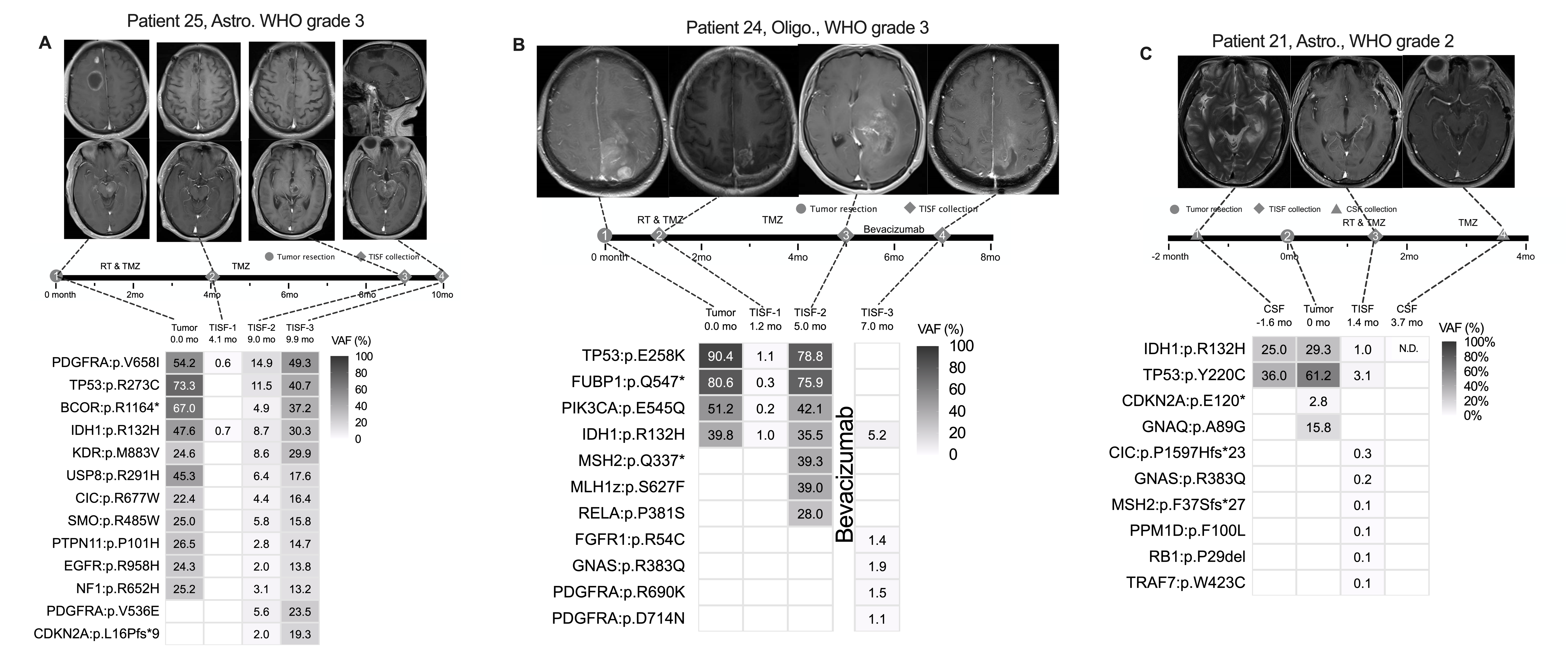

Supplement: Supplementary file 1 — Supporting information [file CTM2-11-e567-s002.tiff]

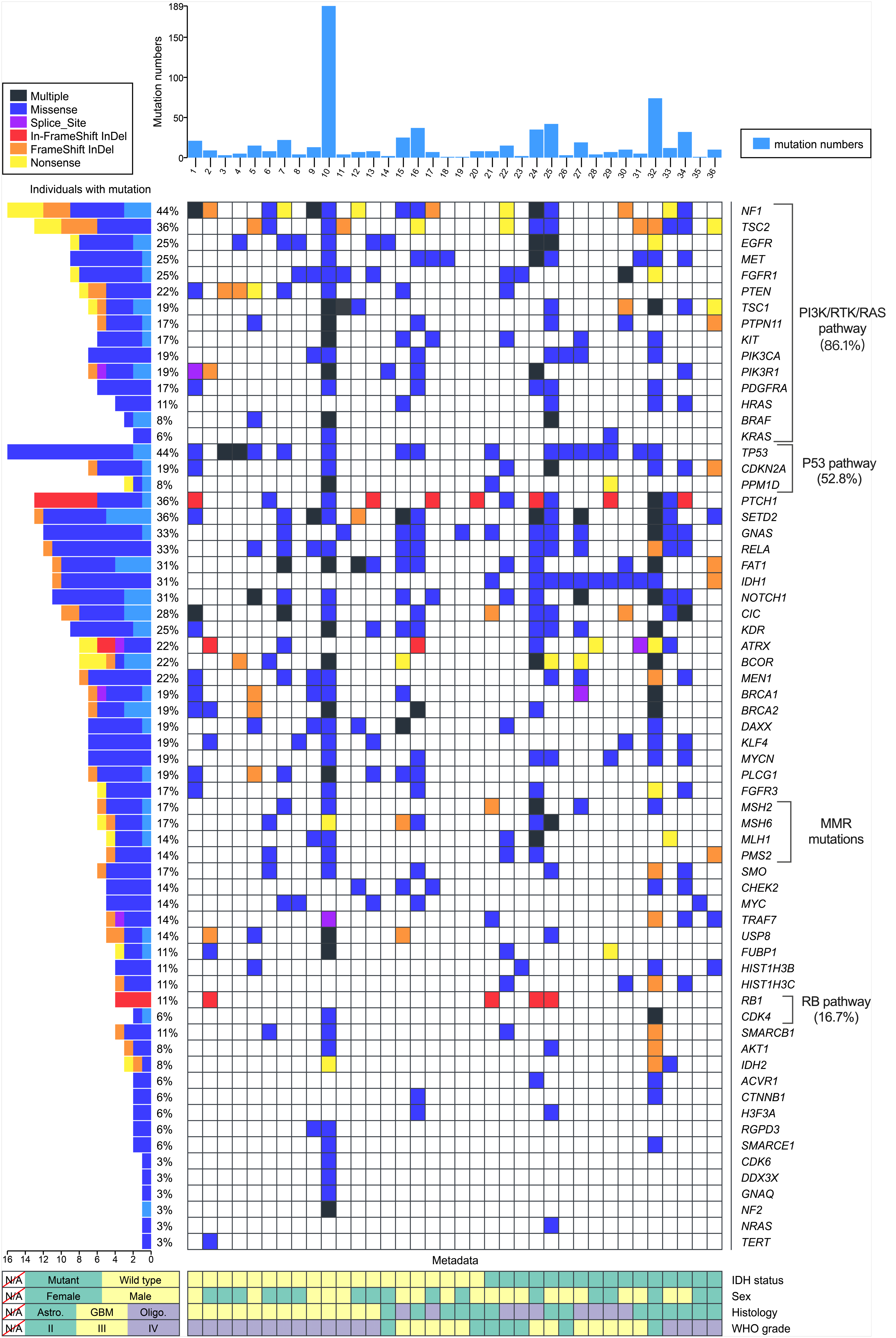

Supplement: Supplementary file 2 — Supporting information [file CTM2-11-e567-s005.tiff]

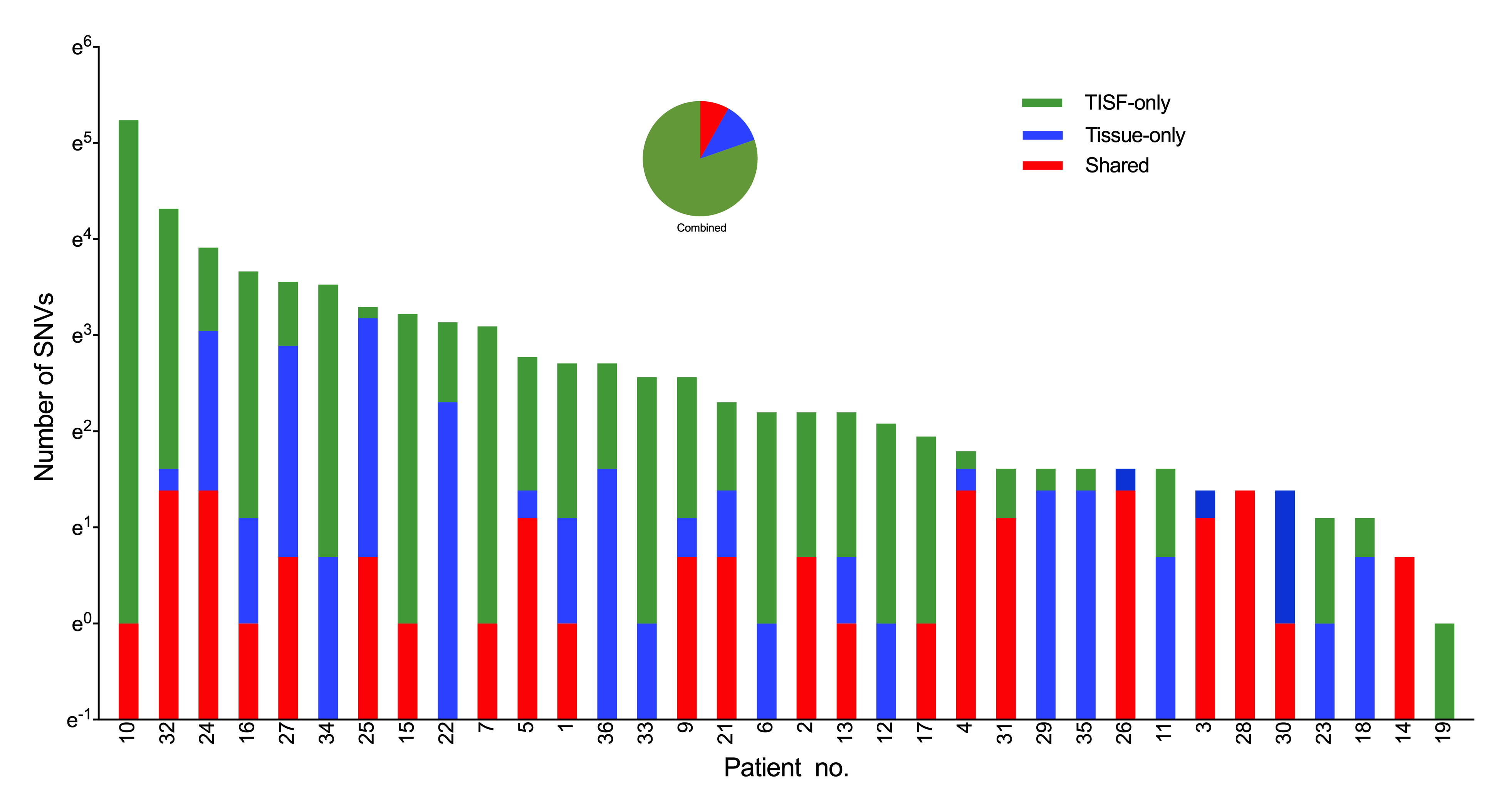

Supplement: Supplementary file 3 — Supporting information [file CTM2-11-e567-s003.tiff]

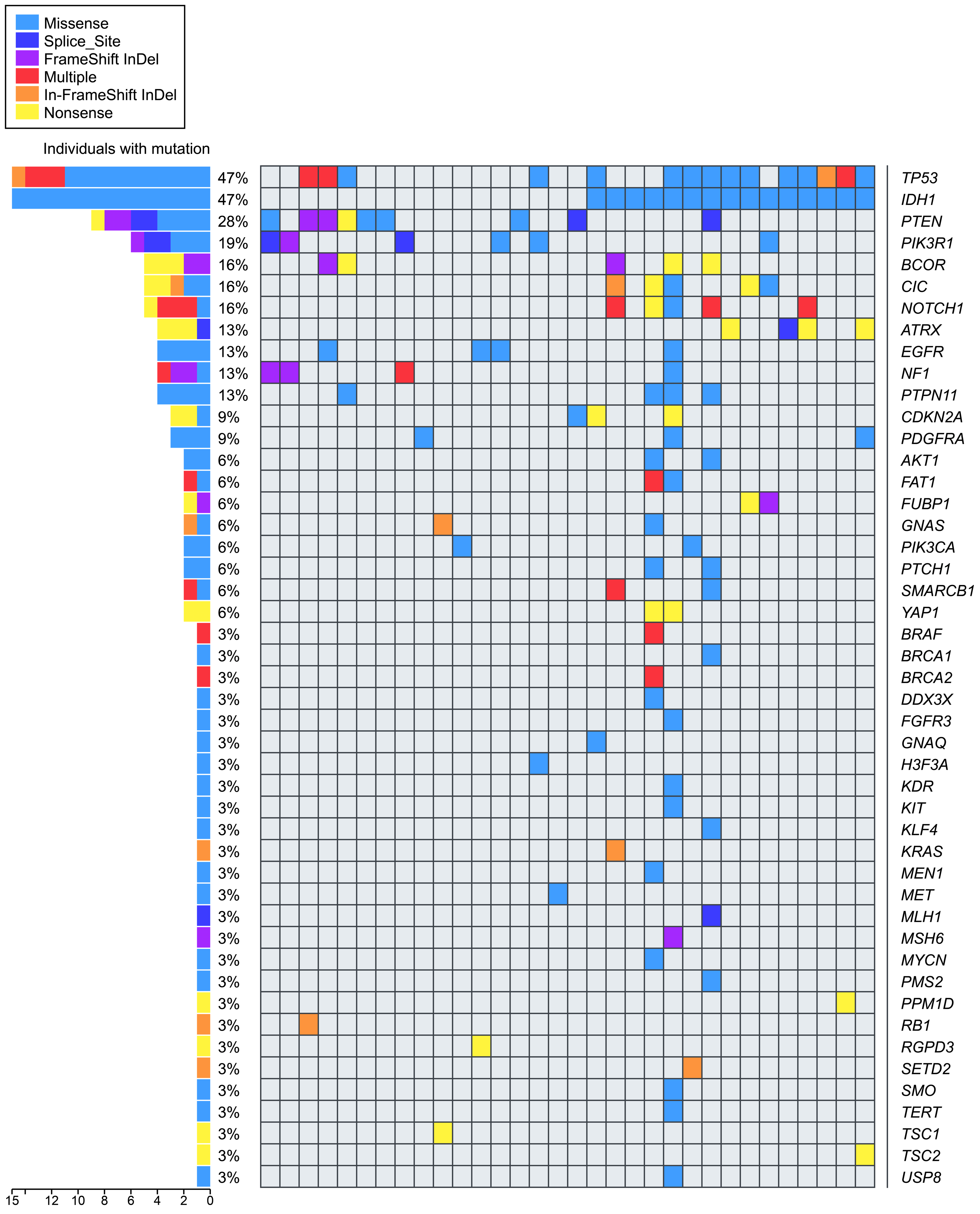

Supplement: Supplementary file 4 — Supporting information [file CTM2-11-e567-s001.tiff]
