# Supplementary material for: Integrating real‐time in vivo tumour genomes for longitudinal analysis and management of glioma recurrence
Source: Clin Transl Med. 2021 Nov 4;11(11):e567. doi: 10.1002/ctm2.567 (PMC8567036; doi:10.1002/ctm2.567)
Supplement: Supplementary file 5 — Supporting information [file CTM2-11-e567-s006.docx]

**Integrating real-time in vivo tumor genomes for longitudinal analysis and management of glioma recurrence**

Zhiyuan Sheng^1,3^, Jinliang Yu^1,3^, Kaiyuan Deng^1,3^, Yage Bu^1,3^, Shuang Wu^1,3^, Sensen Xu^1,3^, Yushuai Gao^1,3^, Qianqian Zhang^1,3^, Zhaoyue Yan^1,3^, Chaojie Bu^2,3^, Zhongcan Chen^2,3^, Jianjun Gu^2,3^, Yan Jia^4,5^, Xinya Gao^4,5^, Ajmal Zemmar^2,3^, Fitri Sumardi^2,3^, Juha Hernesniemi^2,3^, Lingfei Kong^7^, Gang Liu^8^, Ming Li^1,3^, Meiyun Wang^6^, Tianxiao Li^2,3^, and Xingyao Bu^1,3^

^1^Department of Neurosurgery, Zhengzhou University People’s Hospital, Henan Provincial People’s Hospital, Zhengzhou, 450003, China.

^2^Juha International Center for Neurosurgery, Henan Provincial People’s Hospital, Zhengzhou, 450003, China.

^3^Juha International Central Laboratory of Neurosurgery, Henan Provincial People’s Hospital, Zhengzhou, 450003, China.

^4^Department of Neurology, Henan Provincial People's Hospital, Zhengzhou, 450003, China.

^5^Laboratory of Neurology, Henan Provincial People's Hospital, Zhengzhou, 450003, China.

^6^Department of Radiology, Henan Provincial People's Hospital, Zhengzhou, 450003, China.

^7^Department of Pathology, Henan Provincial People’s Hospital, Zhengzhou, 450003, China.

^8^Department of Center for Clinical Single Cell Biomedicine, Clinical Research Center, Department of Oncology, Henan Provincial People's Hospital, The People's Hospital of Zhengzhou University, Zhengzhou, 450003, China

**Supplementary data**

**Table S1: Clinico-pathological characteristics of patients with TISF collection**

| **Characteristic** |  | **TISF (n=36)** | ***P*^b^** |
| --- | --- | --- | --- |
| **Age** (y); median (range) |  | 56.5 (29-79) |  |
| **Sex** |  |  |  |
| Male; number (%) |  | 19 (52.8) |  |
| Female; number (%) |  | 17 (47.2) |  |
| **Histopathology** |  |  |  |
| IDH wild-type glioblastoma; number (%) |  | 13 (36.1) |  |
| Astrocytoma; number (%) |  | 14 (38.9) |  |
| Oligodendrocytoma; number (%) |  | 9 (25.0) |  |
| **WHO grade** |  |  |  |
| 4; number (%) |  | 17 (47.2) |  |
| 2&3; number (%) |  | 19 (52.8) |  |
| **IDH status** |  |  |  |
| wild type; number (%) |  | 20 (55.6) |  |
| mutant; number (%) |  | 16 (44.4) |  |
| **Divided groups**^a^ |  |  |  |
| "Naive"; number (%) |  | 6 (16.7) |  |
| "Stable"; number (%) |  | 14 (38.9) |  |
| "Relapse"; number (%) |  | 16 (44.4) |  |
| **Interval between surgery and first TISF** (m) |  | |  |
| IDH wild-type glioma; median (range) |  | 5.7 (0.3-28.5) | 0.0901 |
| IDH mutant glioma; median (range) |  | 13.8 (0.8-42.4) |  |
| (A)"Naive"; median (range) |  | 0.7 (0.3-1.0) | A-B: <0.0001 |
| (B)"Stable"; median (range) |  | 3.8 (1.2-28.5) | B-C: <0.0001 |
| (C)"Relapse"; median (range) |  | 14.0 (3.2-42.4) | A-C: <0.0001 |
| **Tumor touching CSF on MRI** |  |  |  |
| Non-contact; number (%) |  | 6 (16.7) |  |
| Close contact; number (%) |  | 12 (33.3) |  |
| Dissemination contact; number (%) |  | 18 (50) |  |
| **Extent of resection by MRI** |  |  |  |
| Gross total; number (%) |  | 16 (44.4) |  |
| Residual; number (%) |  | 20 (55.6) |  |
| **Prior therapy** |  |  |  |
| Resection; number (%) |  | 36 (100) |  |
| Radiation; number (%) |  | 21 (58.3) |  |
| Temozolomide; number (%) |  | 30 (83.3) |  |
| Local chemotherapy; number (%) |  | 30 (83.3) |  |
| Bevacizumab; number (%) |  | 5 (13.9) |  |

^a^"naive" referred to postoperative treatment-naive, "stable" to progression-free on MRI, and "relapse" to progression or relapse on MRI.

^b^*P* values were calculated using nonparametric tests for comparison with either Mann Whitney test or Fisher's exact test where appropriate.

**Table S2: Clinico-pathological characteristics of patients with CSF collection**

| **Characteristic** |  | **(+) ctDNA in CSF** | **(−) ctDNA in CSF** | | ***P*^a^** | |
| --- | --- | --- | --- | --- | --- | --- |
| **n=34**^b^ |  | **n=18** |  | **n=16** |  |  |
| **Age** (y); median (range) |  | 52.0 (8-80) |  | 48.0 (28-64) |  | 0.6887 |
| **Sex** |  |  |  |  |  | >0.9999 |
| Male; number (%) |  | 12 (66.7) |  | 10 (62.5) |  |  |
| Female; number (%) |  | 6 (33.3) |  | 6 (37.5) |  |  |
| **Diagnosis by** |  |  |  |  |  |  |
| Image; number (%) |  | 12 (66.7) |  | 3 (18.8) |  |  |
| Resection; number (%) |  | 6 (33.3) |  | 13 (81.2) |  |  |
| **Tumor touching CSF**; number (%) |  | 18 (100) |  | 11 (68.8) |  | 0.0157^b^ |
| **Clinical Stage** |  |  |  |  |  |  |
| (A)Before surgery; number (%) |  | 13 (72.2) |  | 5 (31.2) |  | A-B: 0.0006 |
| (B)Progression-free after surgery; number (%) |  | 0 (0) |  | 9 (56.3) |  | B-C: 0.0048 |
| (C)Progression after surgery; number (%) |  | 5 (27.8) |  | 2 (12.5) |  | A-C: >0.9999 |

^a^*P* values were calculated using nonparametric tests for comparison with either Mann Whitney test or Fisher's exact test where appropriate.

^b^Patient 21 was counted as 2 since he received two times of CSF collection with different ctDNA results.

**Table S3: Comparison between CSF-TISF pairs.**

| **Characteristic** |  | **(+) ctDNA in CSF** |  | **(−) ctDNA in CSF** |  | ***P*^a^** |
| --- | --- | --- | --- | --- | --- | --- |
| **n=11** |  | n=4 |  | n=7 |  |  |
| **Age** (y); median (range) |  | 59 (48-65) |  | 55 (36-64) |  |  |
| **Sex** |  |  |  |  |  |  |
| Male; number (%) |  | 4 (100) |  | 4 (57.1) |  |  |
| Female; number (%) |  | 0 (0) |  | 3 (42.9) |  |  |
| **WHO grade** |  |  |  |  |  |  |
| 4; number (%) |  | 2 (50) |  | 2 (28.6) |  |  |
| 2&3; number (%) |  | 2 (50) |  | 5 (71.4) |  |  |
| **Interval TISF to CSF** (m); median (range) |  | 1.8 (1.0-3.3) |  | 2.3 (1.2-6.1) |  | 0.5636 |
| **Tumor touching CSF space** |  |  |  |  |  | 0.4909 |
| Not contact; number (%) |  | 0 (0) |  | 2 (28.6) |  |  |
| Contact; number (%) |  | 4 (100) |  | 5 (71.4) |  |  |
| **Progression on MRI**; number (%) |  | 4 (100) |  | 1 (14.3) |  | 0.0152 |
| **With malignant cells in TISF**; number (%) |  | 2 (50) |  | 3 (42.9) |  | >0.9999 |

^a^*P* values were calculated using nonparametric tests for comparison with either Mann Whitney test or Fisher's exact test where appropriate.


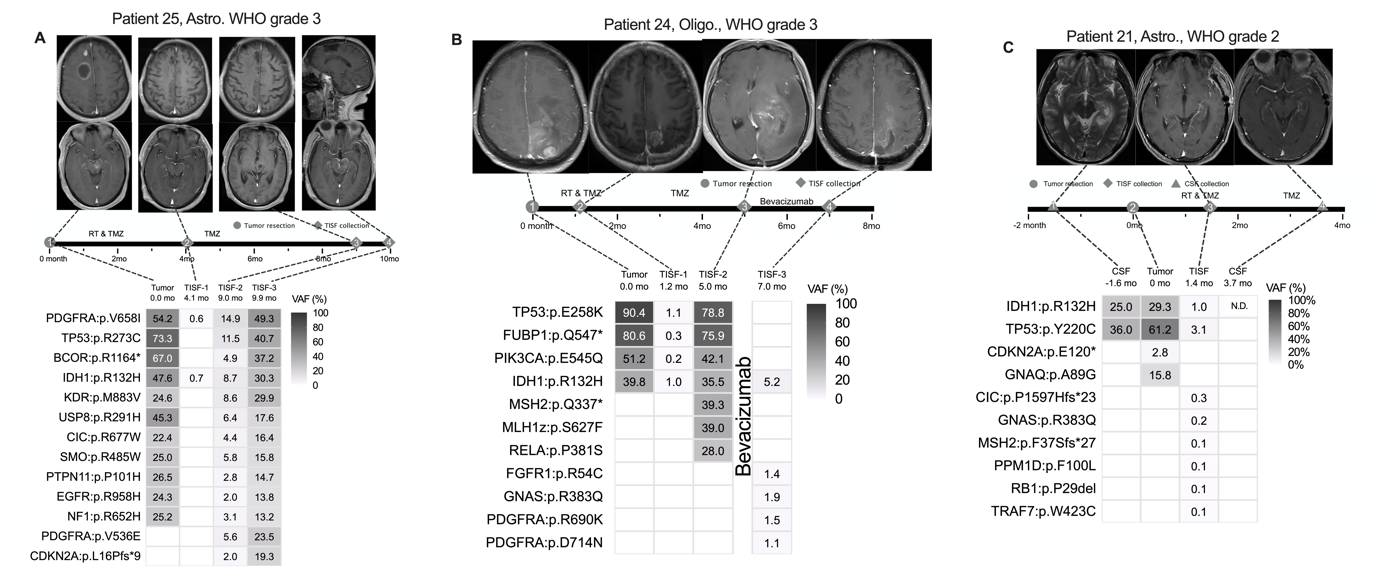


**Figure S1: Collection of representative cases.**

**(A)** and **(B)** are two cases that received multiple TISF collections, both supporting that the increase of VAFs of specific mutations could reflect the tumor progression. Besides, **(B)** shows the temporal beneficialness of Bevacizumab (BEV) for the patient at the molecular as well as the imaging level. **(C)** is a representative case for the comparison of detectability of ctDNA in CSF and TISF. The preoperative CSF detected 2 glioma-derived mutations, but in the postoperative one, ctDNA was not detected, while mutations were detected in the TISF early after surgery. Astro.: astrocytoma. Oligo.: oligodendroglioma. N.D.: not detected.


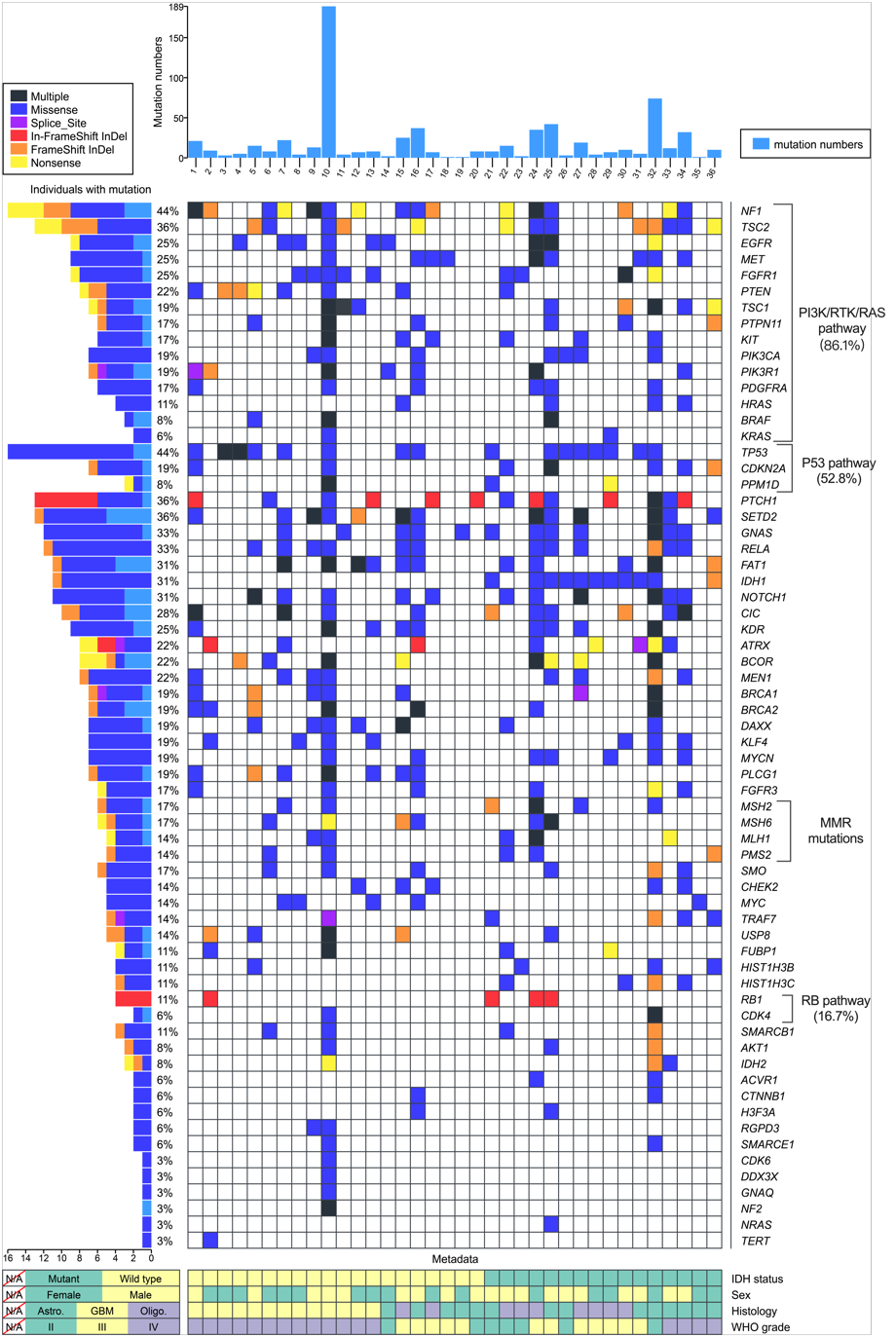


**Figure S2: Mutational landscape of glioma in the TISF.**

Oncoprint of TISF mutations in 36 patients with glioma. Shown were single nucleotide variants (SNVs) which were frequently observed across patients. Astro.: astrocytoma. Oligo.: oligodendroglioma. GBM: glioblastoma. The mutational landscape was generated using the Oviz-Bio^1^.

**
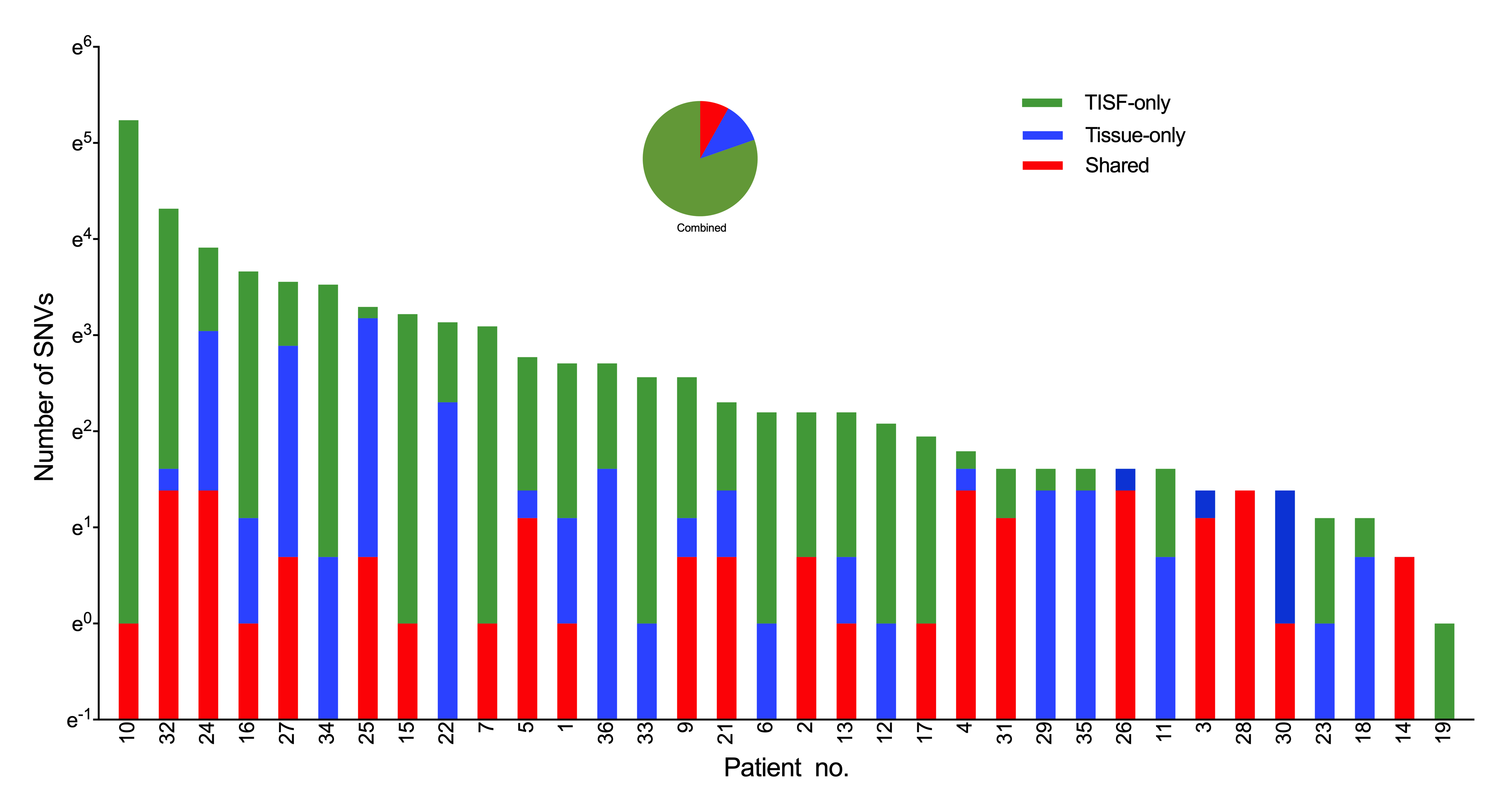
**

**Figure S3:** Frequency of shared versus tissue-only or TISF-only mutations in matched tumor tissue–TISF sample pairs (n=34). The inset showed the aggregate number of mutations for each cohort.


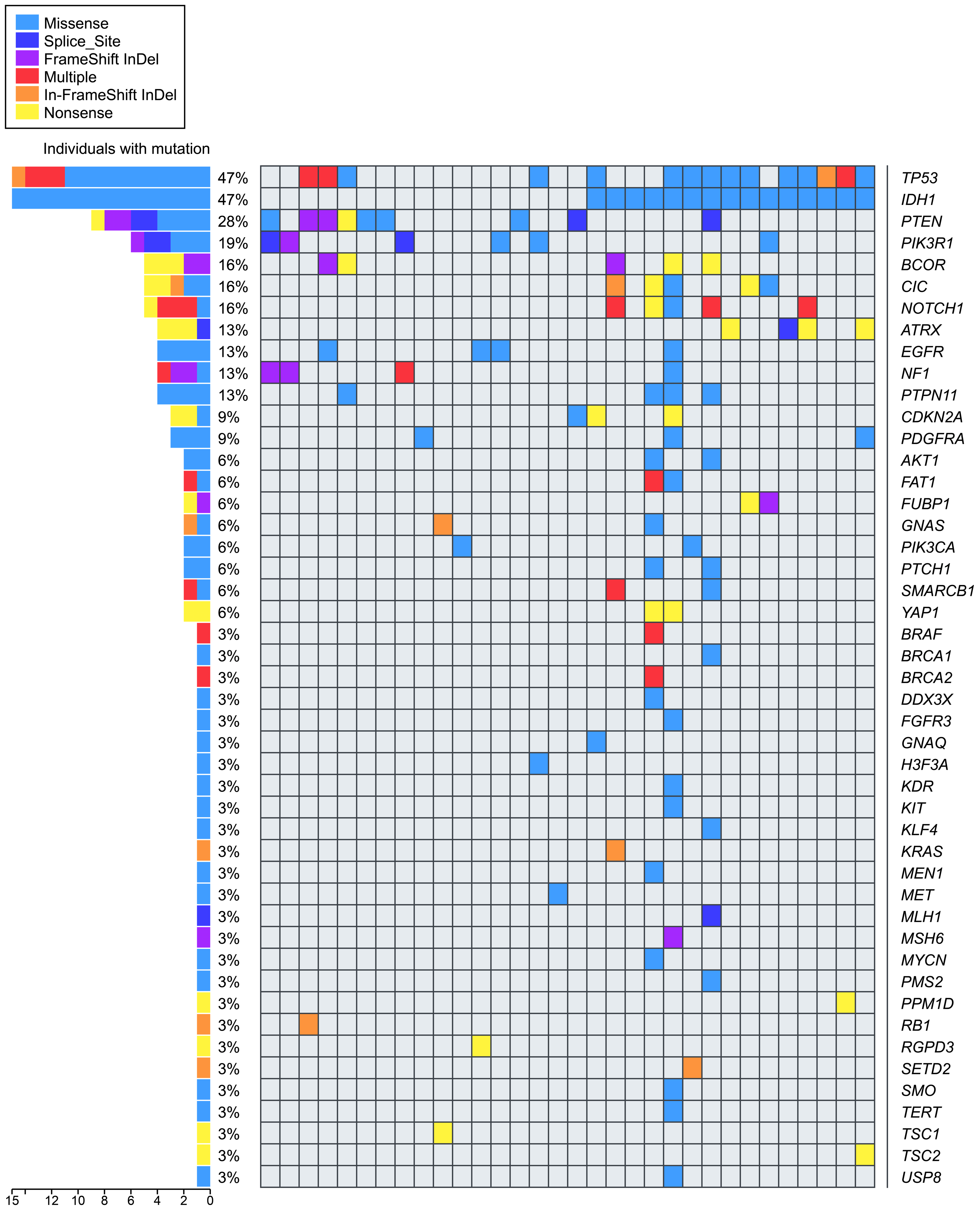


**Figure S4: Mutational landscape of glioma in tumor tissues.**

Oncoprint of mutations in the tumor tissues of 32 patients with glioma. Shown were single nucleotide variants (SNVs) which were frequently observed in the tumor tissues. Columns from left to right refer to patients 1-7, 9-18, 21-32, 34-36 (n=32). Tumor tissues of patients 8 and 20 were not available for sequencing, while any SNV was not detected in the tissues of patients 19 and 33. The mutational landscape was generated using the Oviz-Bio^1^.

**References**

1. Jia, W.; Li, H.; Li, S.; Chen, L.; Li, S. C. Oviz-Bio: a web-based platform for interactive cancer genomics data visualization, *Nucleic Acids Res*. **2020, *48,*** W415-W426.
